# Enamel remineralizing potential of three different agents - A qualitative and quantitative analysis

**DOI:** 10.6026/973206300213587

**Published:** 2025-10-31

**Authors:** Bhavana Badaka, Vijetha Badami, Juveria Masreen, Yoshna Sree V, Sanjana Reddy G, Tarun Teja J

**Affiliations:** 1 Department of Conservative Dentistry and Endodontics, MNR Dental College and Hospital, Sangareddy, Telangana, India

**Keywords:** Dental caries, enamel remineralization, Artificial saliva, nanoparticles, energy dispersive X-ray, vickers hardness number

## Abstract

Dental enamel is susceptible to acid-induced demineralization and while fluoride treatments are effective, they have limitations such as superficial remineralization and risk of fluorosis. This *in vitro* study examined the remineralization potential of calcium phosphate (CaP) nanoparticles, bioactive glass (BAG) nanoparticles and nano-hydroxyapatite (nHAp) on artificially demineralized human enamel. Energy-dispersive X-ray spectroscopy and Vickers microhardness were used to assess the 60 premolars after they were prepared, separated into four groups (control, CaP, nHAp and BAG), demineralized and treated daily for seven days. All nanoparticle groups showed significant remineralization versus control, with nHAp achieving the highest hardness and Ca/P ratio, followed by BAG and CaP. Thus, we show nHAp as the most effective non-invasive remineralizing agent for early carious lesions.

## Background:

The body's most hardest and mineralized tissue is dental enamel [1], protects dentin and pulp from mechanical, thermal and chemical stresses. Composed of over 96% inorganic apatite crystals [2], it is vulnerable to acid-induced demineralization caused by bacterial metabolism of carbohydrates, which lowers pH to 4.5-5, leading to mineral loss [3]. Early enamel lesions are reversible through remineralization [4], which restores partially demineralized hydroxyapatite crystals. Fluoride promotes remineralization by forming acid-resistant fluorapatite [5, 6], but studies show it may cause superficial repair while deeper layers demineralize, leading to lesion progression [7]. To address these limitations, newer agents deliver bioavailable calcium and phosphate directly to lesions for deeper repair. Nano-hydroxyapatite (nHAp) penetrates enamel micropores, replenishing lost minerals and forming a protective enamel-like layer [8]. Bioactive glass (BAG) releases calcium and phosphate ions, forming carbonated hydroxyapatite and sealing dentinal tubules, reducing sensitivity [9]. Calcium phosphate systems like tricalcium phosphate and CPP-ACP act as sustained ion reservoirs, maintaining supersaturation and promoting crystal growth [10]. Nanoparticles offer advantages owing to their high ratio of surface to volume, enhancing remineralization efficiency [11, 12]. Modern dentistry emphasizes non-invasive management of early carious lesions using optimal remineralizing agents, which can reverse initial enamel demineralization and enhance the longevity and aesthetics of restorations. Therefore, it is of interest to describe and compare the remineralization potential of hydroxyapatite (HAp), bioactive glass (BAG) andcalcium phosphate (CaP) nanoparticles on enamel subsurface lesions, with the null hypothesis that all three agents exhibit equal efficacy.

## Materials and Methods:

The study's protocol was authorized by MNR Dental College and Hospital's Ethics Committee (IEC No: MNRDCH/IEC/2022-23/02). The Department of Oral Maxillofacial Surgery at MNR Dental College provided sixty human maxillary premolars that had been extracted for orthodontic purposes. To stop microbiological growth, the recently extracted teeth were preserved in a 0.1% thymol solution. The sample size of sixty was chosen based on studies which were performed earlier [3, 13]. Inclusion criteria required the teeth to be fully developed and structurally sound, without any caries, cracks or restorations. Teeth were excluded if they showed any signs of decay, early enamel demineralisation, surface wear such as erosion, abrasion or developmental anomalies like fluorosis, hypoplasia or resorption. This careful selection aimed to ensure uniformity and reliability of the specimens used for enamel remineralization assessment.

## Specimen preparation:

Sections of the teeth were made 1 mm below the CEJ, using a dual-sided diamond disc and then the crown portions were sectioned in mesiodistal direction to obtain buccal and lingual halves. The buccal halves were embedded in molds made of self-polymerizing acrylic resin, ensuring the buccal surface faced upward. The enamel surfaces were polished and a 4 X 4 mm working window was marked. Nail varnish was applied to the crown's exterior outside of the working window. To ensure hydration and avoid desiccation, all specimens were kept in deionised water at room temperature.

## Demineralisation:

The samples underwent an artificial demineralization process by being immersed in a specially formulated demineralizing solution. A pH of 4.4 was achieved by adding 1 M potassium hydroxide to this solution, which contained 2.2 mM calcium chloride, 2.2 mM sodium dihydrogen phosphate dihydrate and 0.05 M acetic acid. The solution was replenished every 24 hours for 3 days.

## Group allocation and remineralization:

The teeth were then divided into four groups (n = 15):

[1] Group 1: Artificial saliva (control group). This simulated salivary medium was composed of 1.5 mMcalcium chloride, 0.9 mM sodium dihydrogen phosphate dihydrate and 0.15 molar potassium chloride and was maintained at a neutral pH level of 7 to mimic the natural oral environment.

[2] Group 2: Calcium phosphate nanoparticles with average particle size of 30-50 nm with atomic weight of 100.09 g/mol, density of 3.14 g/cm3

[3] Group 3: Hydroxyapatite nanoparticles with average particle size of 20-80 nm with atomic weight of 502.31 g/mol, density of 3.16 g/cm3

[4] Group 4: Bioactive glass nanoparticles with average particle size of <100 nm with density of 1.5 g/cm3.

The remineralizing solutions were prepared by dispersing the respective nanoparticles in deionised water to achieve a final concentration of 10% (w/v). Every solution's pH was meticulously brought down to 7.0, using 2M hydrochloric acid (HCl) in small, controlled volumes to maintain a physiologically relevant and stable environment. The solutions were then stirred to ensure homogenous distribution of nanoparticles; freshly prepared solutions were used for the remineralization procedures. Samples were dried and exposed to the agents and left alone for five minutes every day for seven days. Following that, the specimens were kept in artificial saliva that was replaced daily.

## The four tested materials' capacity for remineralization was assessed using the following criteria:

## X-ray dispersive spectroscopy:

Was used in order to evaluate and compare the composition of calcium and phosphorous chemical weight percentage which establishes the variations in mineral content in demineralized and remineralized areas. Every element was identified by its known wavelength on the X-axis, which was shown on the Y-axis as a peak and peak intensity.

## Vicker's microhardness test:

This establishes how well a material can bear pressure without deforming. For ten seconds, each specimen was loaded with 100g. The final microhardness was determined by taking the average of the three indentations that were noted for each specimen.

## Statistical analysis:

The statistical package for M3 Window (SPSS Inc., Chicago, Illinois, USA) version 22.0 of the Statistical Package for the Social Sciences (SPSS) was used to conduct the statistical analysis. Following demineralization, there were no statistically significant differences between the experimental groups (p = 0.140), confirming the baseline mineral content uniformity. Intragroup differences were evaluated using the Friedman Two-Way ANOVA and Kruskal-Wallis test and intergroup differences were further elucidated through post hoc pairwise comparisons. Improvements in Ca/P ratios were statistically significant for all groups (P < 0.001). To assess enamel surface recovery, the mean comparison of Vickers microhardness test values was carried out. Surface microhardness increased statistically significantly in all groups following remineralization, according to intragroup analysis using paired statistical tests (p < 0.001). A one-way ANOVA was used to compare the mean Vickers microhardness values between groups and post hoc pairwise analysis revealed statistically significant differences between the treatment groups (p < 0.001).

## Results:

After demineralization, all groups-control, calcium phosphate (CaP), nano-hydroxyapatite (nHAp) and bioactive glass (BAG)-had similarly low enamel hardness (around 279-282 VHN) and Ca/P ratios (about 1.53-1.54), indicating comparable mineral loss. Following remineralization, all treated groups showed significant improvements compared to the control (VHN 280.72 ± 5.81, Ca/P ratio 1.53 ± 0.012). nHAp (Group 3) achieved the highest values with a VHN of 426.64 ± 7.83 and Ca/P ratio 1.8650 ± 0.11375, followed by BAG (Group 4) with 408.38 ± 4.37 VHN, 1.7964 ± 0.026 Ca/P and CaP (Group 2) with 391.74 ± 6.29 VHN, 1.7361 ± 0.014 Ca/P. Significant differences between all groups were confirmed by post hoc analysis (p < 0.001) establishing nHAp as the most effective remineralizing agent, followed by BAG and then CaP (Table 1 - see PDF).

## Discussion:

Recent cariology research emphasizes on minimally intrusive management of early, initial carious lesions by using nanoparticle-based remineralizing agents such as nano CAP, nHAP and nano BAG, which maintain ionic supersaturation, promote mineral redeposition and mimic natural enamel while offering enhanced bioactivity and antimicrobial effects [14]. By supplying calcium and phosphate ions that integrate into the hydroxyapatite lattice, these agents encourage remineralization, strengthening the enamel and slowing the progression of lesions [15]. This *in vitro* study assessed enamel remineralization using two key parameters: the calcium-to-phosphorus (Ca/P) ratio and surface microhardness. A balanced Ca/P ratio is critical for the establishment of Calcium and Phosphate phases, with higher ratios favoring hydroxyapatite formation, the most stable and desirable mineral for enamel integrity. Deviations from this ratio suggest incomplete or defective remineralization [16]. The study adopted a baseline Ca/P ratio of 1.5, consistent with enamel's natural mineral content. As a non-destructive indicator of enamel strength recovery, surface microhardness of enamel is a physical property that allows the researcher to evaluate the effects of physical and chemical agents on enamel, dentin and cementum. It has a strong correlation with mineral content [4]. Among the agents tested, nano-hydroxyapatite (Group 3) achieved the highest post-remineralization Ca/P ratio (1.8650± .11375) and Vickers microhardness value (427.64 ± 7.83), showcasing its superior remineralizing efficacy. This was followed by bioactive glass (Group 4), calcium phosphate (Group 2) and the control group (Group 1) respectively, emphasizing that while all agents facilitated remineralization, their effectiveness varied. Similar to our findings, numerous studies have consistently supported the effeciency of nHAp in remineralizing demineralized enamel. Mareddy *et al.* (2025) reported that nHAp significantly improved surface microhardness (SMH) in deciduous enamel, outperforming CPP-ACPF and nearly matching the effectiveness of bioactive glass [17]. Vyavhare *et al.*, Haghgoo *et al.* and Manchery *et al.* also confirmed that nHAp outperformed both fluoride and CPP-ACP in restoring microhardness, due to its biomimetic structure, nanoscale size and strong enamel-penetrating capabilities, these findings support nHAp's ability to closely mimic natural enamel, form a uniform apatite layer and facilitate mineral deposition in enamel subsurface lesions, making it a promising non-fluoride remineralizing agent [18].

Several *in vitro* studies show that nanoparticles like nano-hydroxyapatite, calcium phosphate and bioactive glass (20-100 nm) promote superior enamel remineralization by mimicking natural enamel and releasing calcium and phosphate ions. Mok and Weir *et al.* reported that 10% nHAp and NACP formulations outperformed conventional fluoride agents in enhancing enamel repair [10, 18]. Akbarzade and Vollenweider confirmed BAG's ability to form apatite layers, though its mechanical strength depends on nanoparticle organization. When used in deionized water under controlled conditions, such agents mimic natural remineralization by filling enamel microdefects and restoring minerals [19, 20]. This study uniquely tests these nanoparticles in isolation with artificial saliva, highlighting the influence of particle size, crystallization phase and enamel microstructure on remineralization efficacy. Instead of deeply integrating within the enamel structure like nHAp, bioactive glass (BAG), another powerful remineralizing agent, primarily works by releasing ions of calcium and phosphate that precipitate and form an appetite layer on the surface. According to Satyanarayana *et al.* (2014) and Taha *et al.* (2018), formulations containing BAG considerably raised the hardness and mineral content. Better remineralization results were obtained by using BAG in conjunction with CPP-ACPF. As further evidenced by Rahman *et al.* (2024) BAG proves highly bioactive and effective in both independent and combination therapies for enamel repair [21, 22 and 23]. Promising results have also been observed for nano amorphous calcium phosphate (NACP), which primarily serves as an ion reservoir, delivering phosphate (PO_4_^3-^) and calcium (Ca^2+^) ions to the lesion surface. Afterward, these precipitate as hydroxyapatite, but this process is less controlled and frequently results in superficial mineral deposition instead of ordered crystal regrowth and true integration. ACP's remineralizing action is frequently short-lived and does not achieve the same subsurface penetration as BAG or enamel-like structure as nHAp due to its propensity to agglomerate and its instability in water. Similarly, NACP adhesives improved enamel microhardness, increased mineral recovery and released sustained ions, as shown by Fan *et al.* (2020). Produced citrate stabilized calcium phosphate nanoparticles (CPNPs), which demonstrated their versatility for eroded enamel by restoring hardness and mineral content [24]. These studies highlight the significance of release of ions, its kinetics and stabilization in enhancing enamel remineralization with calcium phosphate-based agents. Despite promising *in vitro* results, current studies face limitations such as short duration, lack of dynamic oral conditions and the use of artificial saliva and fixed 10% concentrations in controlled environments may not fully represent clinical outcomes. The application of nHAP, CPNP and BAG nanoparticles as efficient agents to improve the mineral content and surface hardness of enamel is consistently supported by data. nHAP is the preferred non-invasive fluoride-free method for treating early enamel lesions because of its enamel binding affinity, biomimetic structure and superior microhardness recovery performance when compared to other agents.

## Conclusion:

The calcium-to-phosphorus (Ca/P) ratio and surface microhardness improved in all enamel specimens subjected to the remineralization process. Hydroxyapatite nanoparticles showed the strongest remineralization ability and a notable increase in enamel hardness among all treatment groups assessed.
